# Urban attractors: Discovering patterns in regions of attraction in cities

**DOI:** 10.1371/journal.pone.0250204

**Published:** 2021-04-26

**Authors:** May Alhazzani, Fahad Alhasoun, Zeyad Alawwad, Marta C. González

**Affiliations:** 1 Center for Complex Engineering Systems at KACST and MIT, Riyadh, Saudi Arabia; 2 Center for Computational Engineering, Massachusetts Institute of Technology, Cambridge, Massachusetts, United States of America; 3 Department of City and Regional Planning, University of California Berkeley, Berkeley, California, United States of America; Univ. Lyon, ENTPE, Univ. Gustave Eiffel, FRANCE

## Abstract

Understanding the dynamics by which urban areas attract visitors is important in today’s cities that are continuously increasing in population towards higher densities. Identifying services that relate to highly attractive districts is useful to make policies regarding the placement of such places. Thus, we present a framework for classifying districts in cities by their attractiveness to daily commuters and relating Points of Interests (POIs) types to districts’ attraction patterns. We used Origin-Destination matrices (ODs) mined from cell phone data that capture the flow of trips between each pair of places in Riyadh, Saudi Arabia. We define the attraction profile for a place based on three main statistical features: The number of visitors a place received, the distribution of distance traveled by visitors on the road network, and the spatial spread of locations from where trips started. We used a hierarchical clustering algorithm to classify all places in the city by their features of attraction. We discovered three main types of Urban Attractors in Riyadh during the morning period: *G*lobal, which are significant places in the city, *D*owntown, which contains the central business district, and Residential attractors. In addition, we uncovered what makes districts possess certain attraction patterns. We used a statistical significance testing approach to quantify the relationship between Points of Interests (POIs) types (services) and the patterns of Urban Attractors detected.

## Introduction

According to the United Nations’ 2018 World Urbanization Prospects report, 55% of the planet’s population now lives in an urban area and expects the proportion to increase to 68% by 2050 [1]. The density of cities brings economic productivity, provides cultural amenities, and facilitates sustainability. It is also the root of problems related to congestion, health, and safety. A pressing need, in complex and congested cities is a deeper breakdown and understanding of the major flows of people in a city. Understanding how different places in the city influence human mobility is important for urban planning and transportation operations. Furthermore, it extends to other domains such as epidemiology where the way people move has a significant impact on the prevalence of pandemics as is the case in COVID-19 nowadays [2]. Cities are self-organized, complex, adaptive systems, thus, understanding their dynamics is critical for improving them and the lives of their billions of residents. While there is a rich tradition of research in this area, work has generally been hindered by lack of data.

The rise of ubiquitous mobile computing and its ability to passively collect and extract knowledge from large data regarding human mobility promises sweeping change. Today, with the ubiquity and pervasiveness of technology, data generated from mobile phones enable data scientists to better understand the various aspects of the behavior of individuals, including their mobility patterns [3, 4]. In the human mobility literature, researchers investigate various methods of estimation of the Origin-Destination (OD) matrix. The OD entries at *i*,*j* represent trips originating from a location *i* traveling to location *j*. ODs are very useful for the simulation of flows of people in the state of the art 4-step model [5]. In this paper, we are set to explore the attractiveness of regions in a city by analyzing the ODs generated for the city of Riyadh using massive passive phone calling data.

In this work, we present a novel computational framework for classifying urban places by their attraction patterns. We refer to places on the level of the spatial polygons defined by neighborhood boundaries called Traffic Analysis Zones (TAZ) that constitute the sources and destinations in the OD matrix. We define attraction profiles in terms of statistical features of incoming trips on a given time window. Different places in the city attract visitors differently, as we are set to show later in the paper. We aim to identify patterns of attraction of places based on three main dimensions: how many visitors a place receives, where visitors are coming from, and how long visitors are traveling to reach that place. Furthermore, we investigate if POIs can explain why such patterns emerge. To accomplish that, we used statistical significance testing to relate the decomposition of POI types (services) and the discovered attraction patterns. This information is useful to relate types of businesses and attraction profiles. The analysis is even more impactful in our case study, the city of Riyadh, Saudi Arabia, where a large metro project is being developed and promised to run in early 2021 [6].

The main contribution of this work is as follows:

- We present a computational framework for detecting attraction patterns and further relating POI types to each pattern of attraction.
- We discover three patterns of attraction profiles; Global, Downtown, and Residential attractors. Each has a distinctive signature of spatial dispersion of trip origins, the distribution of distances traveled by visitors through the road network, and the total number of trips.
- We investigate and show how the types of POIs in a region correlate with its attraction profile with varying degrees using a statistical significance testing approach.

## Related work

The standard approach to categorize urban areas classifies regions by their physical features and land use (i.e., commercial, educational, …etc.). Researchers developed models to infer such classifications from phone data [7]. Existing work considers the dynamics of human mobility and flows to classify regions. For example, Yuan *et al.* proposed a topic modeling approach to classify districts into functional zones according to people’s socioeconomic activities mined from taxies and public transport traces and points of interests (POIs) data [8]. Pan *et al.* proposed a land use classification approach based on the social functions of districts also analyzed from GPS taxi traces where districts witness change of land use class dynamically [9]. Toole *et al.* [7] analyzed cell phone data to test cell phone activity patterns to classify land use types. Less is known about how phone data can be used to classify urban regions based on how attractive they are to different origins.

Multiple studies used human mobility behavior to classify urban areas. A study investigated the relationship between land use and mobility [10, 11]. The authors showed that the purposes of people’s trips are strongly correlated with the land use of the trip’s origin and destination. The availability of dynamic data sources allowed for dynamic segmentation of the city according to human mobility behavior. Some studies combined human mobility with land use or POIs data to segment districts in urban areas according to their functions or use. The type of data used to capture human mobility behavior varied between individual GPS traces [12, 13], taxi pickup/drop off locations as in [9, 14], Call Detail Records (CDRs) as in [7, 15], social media check-ins as in [16–18], and bus smart card data as in [19].

Survey travel data has been used to discover the centers (significant places) of a city [20, 21]. A study proposed a method for measuring the centrality of locations that incorporates the number of people attracted to the location and the diversity of activities in which visitors engaged [20]. The proposed method was tested on survey travel data in Singapore to identify the functional centers and track their significance over time. A similar approach focused on analyzing the aggregate behavior of the population to predicted highly attractive events such as the times square during new years eve in New York [22]. Our method is based on validated Origin Destination (ODs) matrices mined from massive cell phone data that captures human mobility. More significantly, our approach incorporates not just the amount of people a place attracts but also where they come from and the road distance they traveled.

Network analysis methods were used to detect hotspots based on flow patterns between locations [15, 23]. A paper used ODs extracted from cell phone data to show how different cities have different mobility behavior signatures based on four main types of movements within the city: between hotspots, to hotspots, originating at hotspots, and random flows [15]. They showed how different cities have different mobility signatures. Additionally, a study used Taxi drop off/pick up traces in Shanghai to create a network of flow between places. They applied community detection to extract sub regions and analyze the interaction between and within sub regions. They found that urban sub-regions have larger internal interactions, while suburban centers are more significant on local traffic. This work made the breakdown of flow patterns instead of the impact of the place in attracting visitors, which is our aim in this paper.

Researchers adapted modeling approaches from Natural Language Processing (NLP) in identifying functional zones in urban areas [8, 24]. One study applied a Latent Dirichlet Allocation (LDA) model on Foursquare check-ins to detect local geographic topics that indicate the potential and intrinsic relations among the locations in accordance with users’ trajectories. Additionally, a study used LDA and POIs to detect functional zones [8]. Our work is different, where we aim to analyze the attraction behavior of a place using measures that have not been explored in any previous work.

## Urban attractors framework

**Fig 1:**
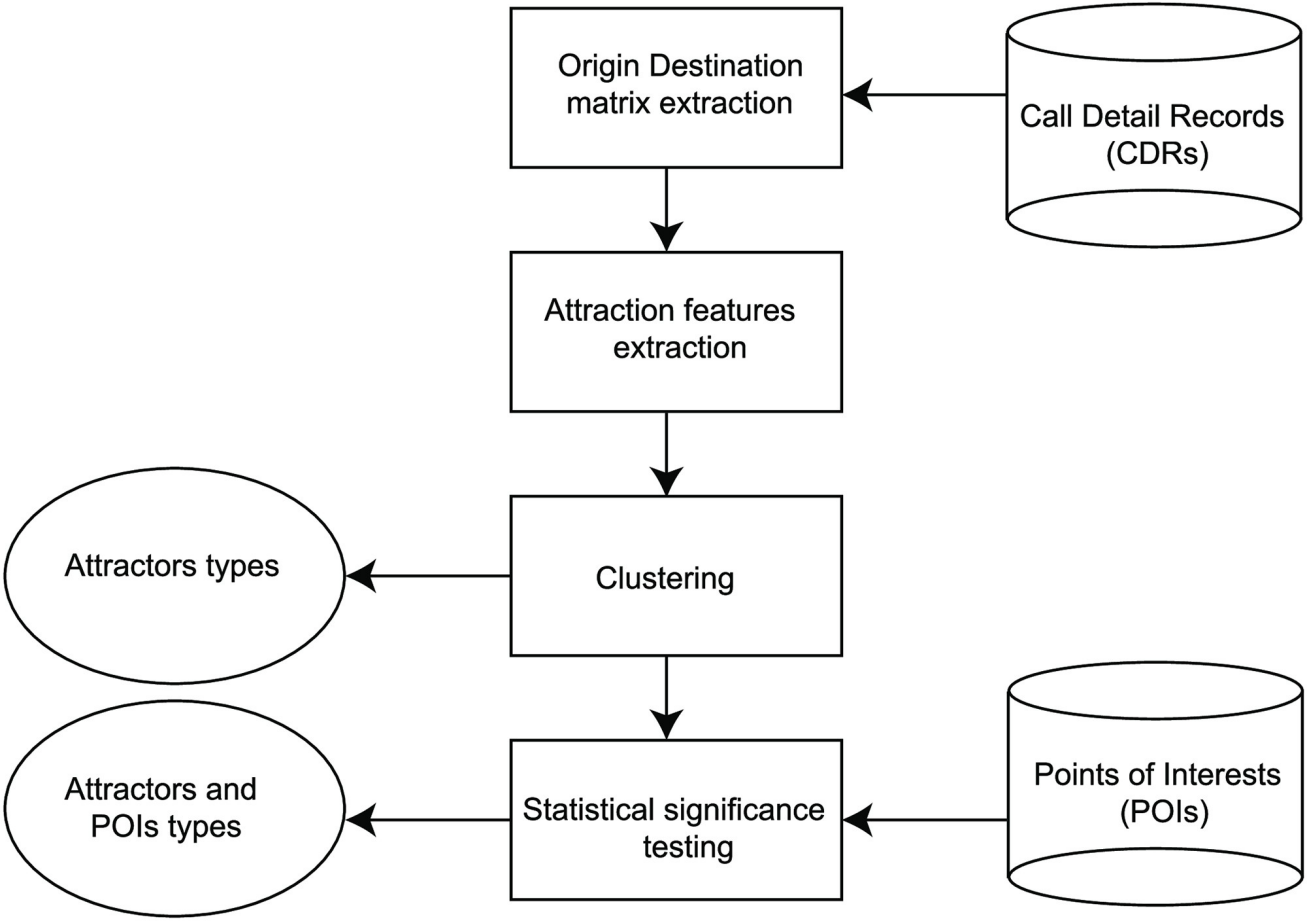
Urban attractors classification framework. It shows all sub-processes to identify attractor profiles. It relies on two data sources: Call Detail Records (CDRs) and Points of Interests (POIs) to detect attractors and relate types of places (POIs) to each attractor pattern.

Fig 1 shows the general structure of the process of identifying attraction patterns in cities with the input datasets and the outputs. The first step in the process is to extract trips from aggregated Call Detail Records (CDRs) of cell phones using state of the art validated origin-destination extraction algorithm described in [25] (described in the Appendix). We use the ODs as a data source for estimating human mobility, where it provides the number of trips from each pair of origin and destination. From the ODs, we mine three statistical features that quantify how attractive a district is: the number of trips a place receives, the spatial dispersion of the origins of all incoming trips, and the distribution of distances visitors traveled to visit the place on the road network. Using classical clustering methods on the attraction features, we classify all regions in the city according to their attraction behavior. Finally, using a statistical significance testing approach, we relate each type of POIs that are significantly concentrated in each type of attractors identified. In the methods section, we explain each process in detail.

## Materials and methods

### Attraction features

We identified statistical features that capture patterns of region attraction from the OD matrix flows between regions in the city. A fundamental feature is the total amount of inflow (visits) a region receives, as the more visitors a place receives, the more attractive it is. Additionally, a more interesting and useful feature of attractiveness is the spread of origins of visitors. A place is more attractive if it attracts visitors from various places in the city, such as universities and hospitals, which attract visitors from all over the city. Thus, the second feature measures how spatially dispersed the origins of trips are. Another useful feature to measure attraction is from the distances traveled by visitors to reach a destination. Longer distances traveled to reach a destination indicate strong attractiveness of that destination. In the following sections, we describe each attraction feature in detail.

#### Inflow

The number of visitors a place receives is the strongest indicator of how attractive the place is. This feature measures the attraction force of a location, where locations that have high inflow (number of visitors) are major attractors in the city. Fig 3*A* shows the distribution of the number of TAZes according to their inflow amount. The majority of TAZes have small to moderate inflow. However, there are a few TAZes that have very large inflows, which makes them highly significant.

The inflow magnitude of a TAZ *i* is calculated from the OD matrix as follows:

$$\begin{array}{c} Inflow_{i}=\sum_{j=1}^{n}T_{ji}, \end{array}$$ (1)

where *n* is the total number TAZes, and *T*_*ji*_ is the number of trips from TAZ *j* to TAZ *i*.

#### Spatial dispersion

**Fig 2:**
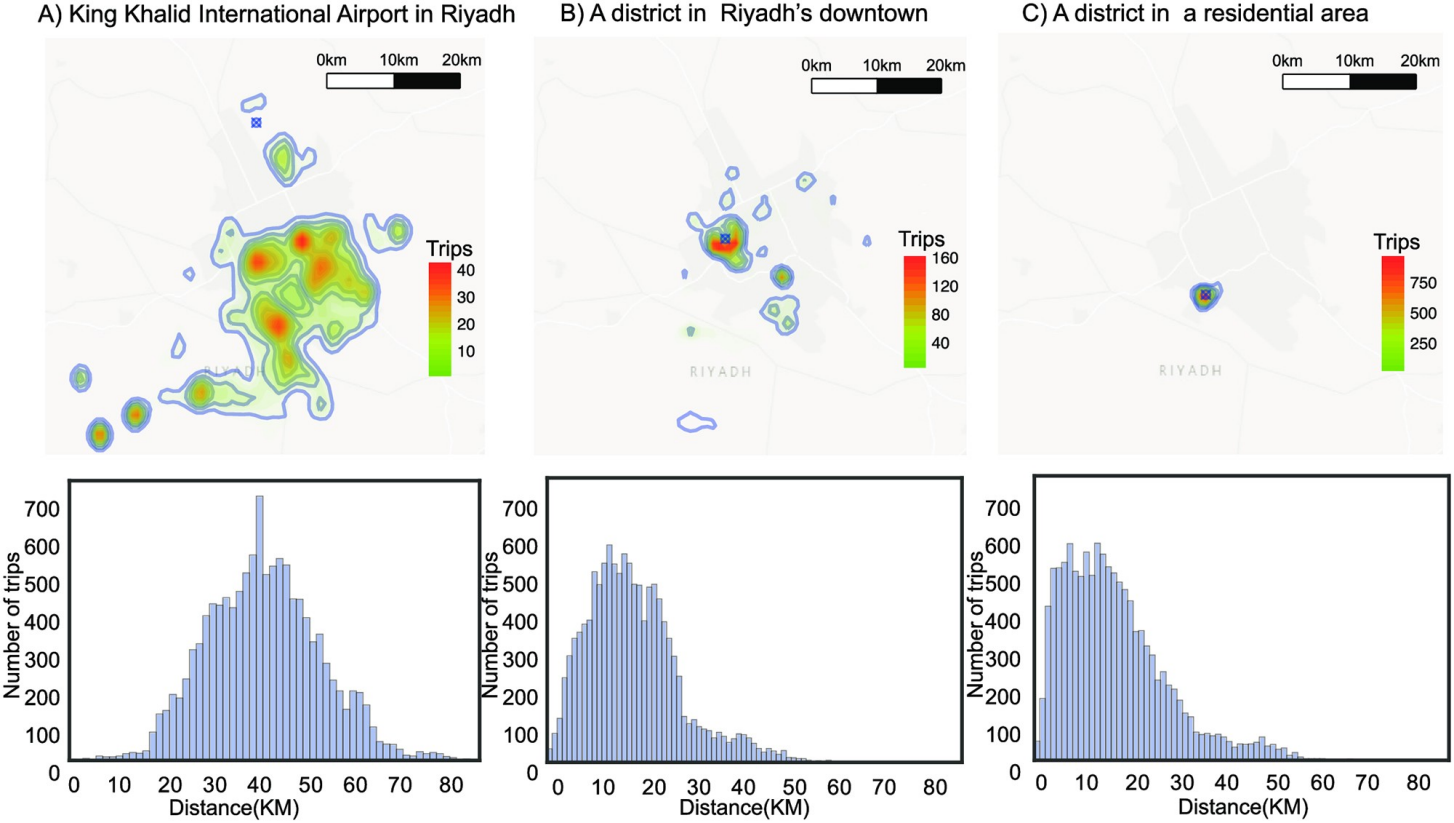
Spatial dispersion and distance distributions of three examples of types of attractors (marked by ⊗). (A) The international airport in Riaydh. (B) A place in the downtown area. (C) A place in a residential area. The top row shows heatmaps of the origins of the inflow, where the color differs corresponds by the amount of trips from that location. The bottom row is the distribution of road distance traveled by visitors of the selected place. The base map was obtained from the U.S. Geological Survey maps.

**Fig 3:**
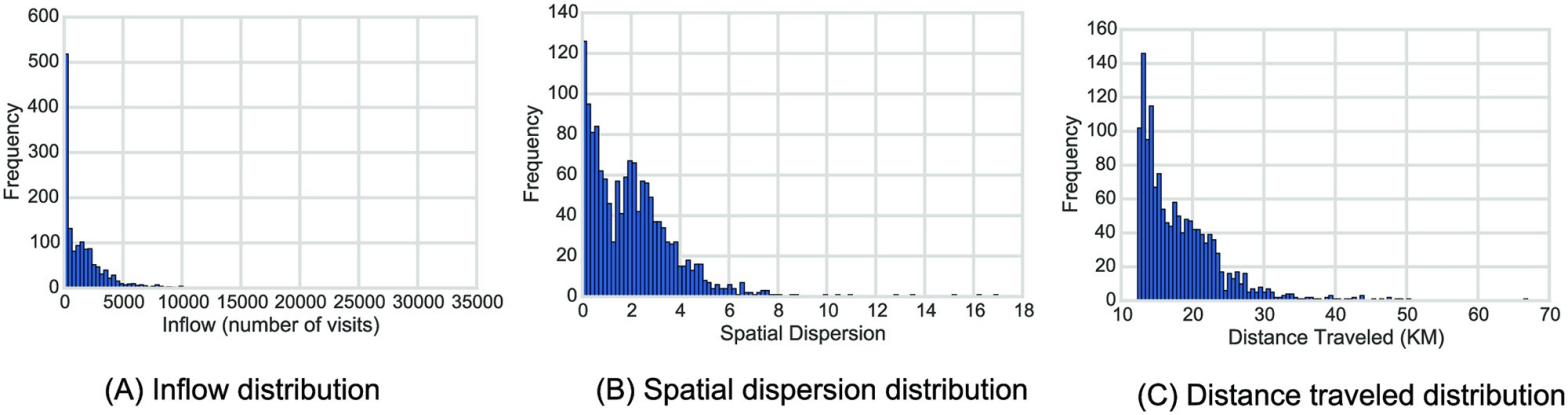
The distribution of attraction features. (A) shows the total inflow received by TAZes in Riyadh. The majority of places receive low to medium inflow, while a few places receive very high inflow, making them highly attractive. (B) Shows the distribution of spatial dispersion, and (C) shows the distribution of distances traveled on the road network.

An informative feature to measure the attraction of a place is to measure the spatial dispersion of the origins of trips it attracts. The spatial dispersion quantifies how spatially dispersed the locations of the origins of trips are in relation to the center of mass of all origins. A place is more attractive if it attracts visitors from various and spread-out places in the city. Major attractors tend to attract people from all over the city (large spatial dispersion), like example *A* in Fig 2. In contrast, less significant attractors only attract people nearby (presenting small spatial dispersion), as in example *C* in Fig 2. Fig 3*B* shows the long tailed distribution of spatial dispersion of TAZes in the city, where the majority of districts have small spatial dispersion, while there are few districts with large spatial dispersion indicating their attractiveness.

We compute the spatial dispersion *SD*_*i*_ of TAZ *i* as follows:

$$\begin{array}{c} SD_{i}=\frac{\sqrt{\sum_{i=1}^{n}w_{i}{\left(X_{i}-X_{c}\right)}^{2}+\sum_{i=1}^{n}w_{i}{\left(Y_{i}-Y_{c}\right)}^{2}}}{\sum_{i=1}^{n}w_{i}}, \end{array}$$ (2)

where *n* is the total number TAZes, *X*_*i*_ and *Y*_*i*_ are the spatial coordinates of the origin of a trip *i*, *w*_*i*_ is the amount of inflow from source TAZ *i*, and *X*_*c*_,*Y*_*c*_ are the coordinates of the spatial center of mass of all origins of all the incoming flow calculated as follows:

$$\begin{array}{c} X_{c}=\frac{\sum_{i=1}^{n}w_{i}.X_{i}}{\sum_{i=1}^{n}w_{i}},Y_{c}=\frac{\sum_{i=1}^{n}w_{i}.Y_{i}}{\sum_{i=1}^{n}w_{i}} \end{array}$$ (3)

#### Distance distribution

Another characteristic that defines attraction patterns is the distribution of distances traveled by visitors. Trips’ distances from each TAZ source to the centroid of the destination TAZ were calculated on the road network of Riyadh. We used the Dijkstra shortest path algorithm [26] to find the optimal routes between all of the origin-destination pairs. This provides a more accurate estimation than using Euclidean or Manhattan distances, as it accounts for the variation in the geometry of the road network in the city.

The bottom row in Fig 2 shows the distance distributions of three examples of districts with different attraction behaviors. In Fig 2*A* the distance distribution to the airport is unique, with a long mean distance (around 40 km.) and a shift in the distribution due to the airport’s distant location in the very far north of the city. On the other hand, Fig 2*B* has an intermediate length of the mean distance with a distribution tail that corresponds to the long distance traveled by some visitors to reach downtown. Finally, a district with low attraction behavior, as in Fig 2*C*, receives trips with short distances. Clearly, for different types of attractors, the distance distributions differ. Thus, we select the mean and the standard deviation of the distribution as features to distinguish attraction behaviors.

We compute the distance distribution’s mean *μ*_*i*_ and standard deviation *σ*_*i*_ of visitors to TAZ *i* as follows:

$$\begin{array}{c} \mu_{i}=\frac{\sum_{u=1}^{m}d_{u}}{m},\sigma_{i}=\sqrt{\frac{\sum_{u=1}^{m}{\left(d_{u}-\mu_{i}\right)}^{2}}{m}}, \end{array}$$ (4)

where *m* is the number of visitors to TAZ *i*, and *d*_*u*_ is the distance traveled by user *u* on the road network.

### Clustering

To discover attractor patterns within cities, regions are clustered using the attraction features discussed in the previous section. We used a Hierarchical Agglomerative Clustering (HAC) approach to categorize all 1492 TAZes in Riyadh based on their attraction features. HAC clusters objects based on their feature vectors. Here, a vector *x*_*i*_ represents the attraction features that describe TAZ *i* as follows:

$$\begin{array}{c} x_{i}=\left[inflow_{i},SD_{i},\mu_{i},\sigma_{i}\right], \end{array}$$ (5)

where *inflow*_*i*_ is the inflow magnitude of TAZ *i*, *SD*_*i*_ is the spatial dispersion of the inflow sources for TAZ *i*, *μ*_*i*_ is the mean of the distances traveled to TAZ *i*, and *σ*_*i*_ is the standard divination of the traveled distance distribution.

HAC uses a bottom up approach by merging similar items to form clusters. Thus, it requires defining how to merge clusters and how to measure the distance between them. We used the complete-linkage algorithm, which merges two clusters based on their most dissimilar objects a follows:

$$\begin{array}{c} D\left(X,Y\right)=\max_{x\in X,y\in Y}d\left(x,y\right), \end{array}$$ (6)

where *d*(*x*,*y*) is the distance between two objects *x* ∈ *X* and *y* ∈ *Y*, and *X* and *Y* are the two sets of clusters. Complete-linkage is conservative when merging clusters; thus it tends to find more compact clusters, which fits our objective in finding closely related attraction patterns. For measuring the distance between clusters’ objects *d*(*x*,*y*), the correlation distance metric is used, which is equivalent to the centered cosine distance, defined as follows:

$$\begin{array}{c} d\left(x,y\right)=1-\frac{\left(x-\bar{x}\right).\left(y-\bar{y}\right)}{{∥\left(x-\bar{x}\right)∥}_{2}{∥\left(y-\bar{y}\right)∥}_{2}}, \end{array}$$ (7)

where $\bar{x}$ and $\bar{y}$ are the mean of the elements of vector *x* and *y* correspondingly. Correlation distance enables finding clusters with unbalanced sizes, which fits our application. We expect to have a small number of places behaving very uniquely as strong attractors and a larger number of places that are not as attractive. Additionally, the correlation score can correct any scaling within a feature, while the final score is still tabulated. Thus, different features that use different scales can still be used.

**Fig 4:**
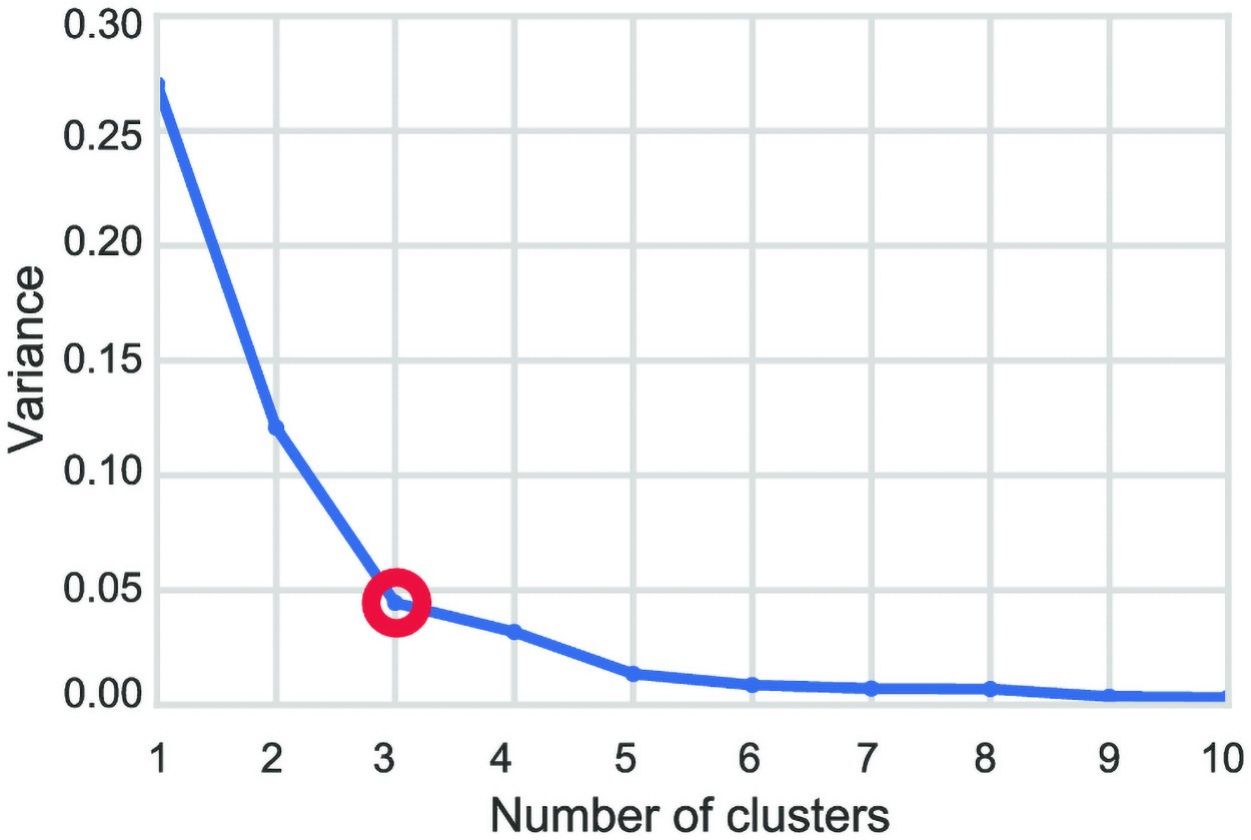
The ratio of the within-cluster variance to the total variance for each possible choice of *K* (number of clusters). The variance decreases as the data is split into more clusters until it stops decreasing significantly at *k* = 3 (marked by a red circle).

HAC provides a hierarchy structure of the classified regions in the city. To determine the number of clusters *k* that best divide the data, we calculate the ratio of the between-cluster variance to the total variance for each possible *k* from 1 to 10. The variance drops as *k* increases until it stops decreasing significantly. We select the *k* that corresponds to the point where the variance stops decreasing significantly, which is *k* = 3 in our case, as shown in Fig 4. The classification process over all TAZes in the city of Riyadh finds three types of attractors that have distinctive features. The following section extends these findings to interpret the results further.

### Statistical significance testing

To interpret what makes a region possess certain attraction behavior, we relate the decomposition of POI types of districts to the different attraction patterns discovered. Using only counts of POIs per attractor type is not sufficient, as the distribution of POI types is highly unbalanced. For example, restaurants are very common and spatially distributed, whereas universities are sparse and only present in a few districts. Thus, we adopted a statistical significance testing approach to rigorously quantify the significance of a POI type to districts with common attraction patterns. The statistical significance testing method measures the probability of observing the amount of a POI type in a spatial zone or larger by chance. It factors in the amount of all POIs in the spatial zone in addition to the amount of POIs with that type tag in the whole city.

We used Fisher’s Exact Test (FET) to relate each type of POIs to the different attractor types. We selected FET because it works for small observations and calculates the exact probabilities rather than approximations as in the Chi-square test. FET aims to test the dependency between two categorical variables given the observed data. In our context, the first variable *POI*_*Type* = *t*_ is the number of POIs that belong to a specific type *t*, and the second variable *POI*_*Zone* = *z*_ is the number of POIs in a spatial zone *z*. FET tests the significance of the overlap *POI*_*Zone* = *z*_ & *POI*_*Type* = *t*_, which is the amount of POI *t* in zone *z*. It quantifies the probability (p-value) of observing that amount of POI type *t* or larger in zone *z* by chance. The smallest the p-value is, the more significant the concentration of POI type *t* in zone *z* is. FET calculates the p-value *p* as follows:

$$\begin{array}{c} p=\frac{(a+b)!(c+d)!(a+c)!(b+d)!}{a!b!c!d!n!} \end{array}$$ (8)

where *a* represents the overlap, which is the amount of POIs of type *t* that are located in zone *z*, *b* represents the amount of POIs of type *t* that are not located in zone *z*, *c* is the amount of other POI types located in zone *z*, *d* represents the number of all the rest of POIs in the city, and *n* is the total amount of all POIs in the city.

FET incorporates two crucial factors when measuring the significance. First, it factors in the amount of other POI types in zone *z* as a measure of purity. If a zone has a large amount of POIs of type *t*, but it also has many other POIs types, that makes type *t* less significant due to this impurity in the decomposition of all POIs in that zone. Second, the significance depends on the amount of POIs of type *t* that are not in the tested zone *z* as a measure of rarity. If there is a large number of POIs of type *t* elsewhere, that makes type *t* insignificance in the tested zone. These two properties make FET superior to trivial methods like calculating the percentages of POI types in spatial zones.

## Results

### Urban attractors

**Fig 5:**
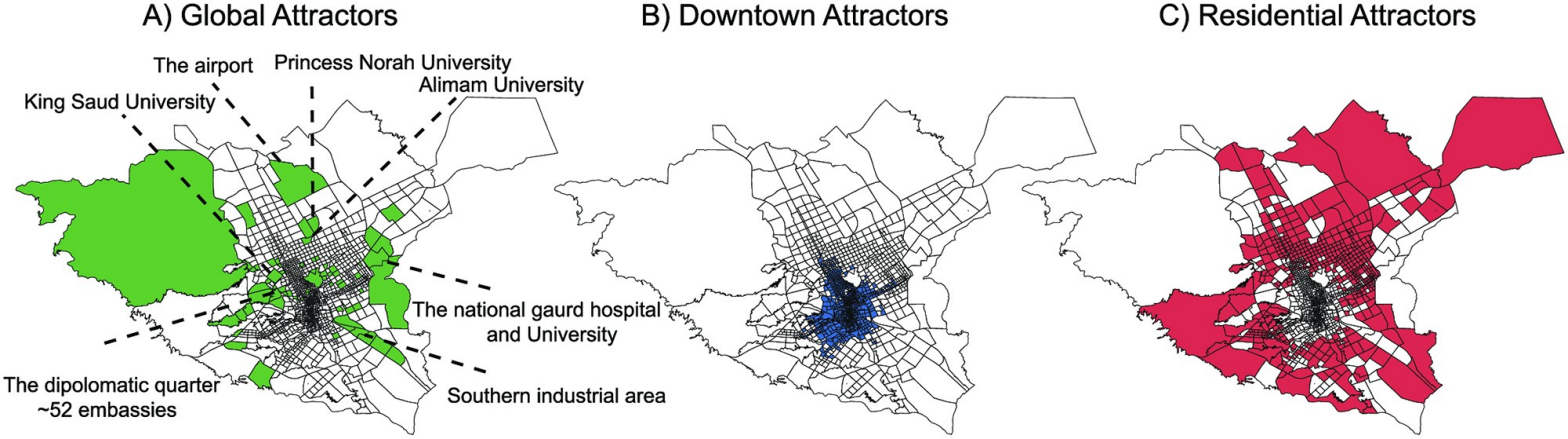
Types of attractors. (A) Global at tractors, which are places with a large number of visitors from all over the city traveling long distances to visit them. These include unique places in the city like the airport, large universities, hospitals, industrial areas, and the diplomatic quarter as annotated on the figure. (B) Attractors with a large number of visitors due to their central location (downtown) are more accessible, and thus the distance visitors travel to reach them is shorter. (C) Less attractive places that are located on the outer (residential) areas of the city.

Using the proposed clustering framework and the attraction features, we discovered three main types of urban attractors in Riyadh based on distinguishable attraction profiles. Fig 5 shows the three types of attractors detected, where the polygons are the TAZes. The global attractors are the ones that have significant influence on the whole city, hence their name. Unlike the remaining attractor types, the locations of these attractors seem to be random around the city, which indicate their uniqueness in services and places they offer. The second detected type is the downtown attractors, which play a significant influence, after global, to attract trips. These are mostly clustered in the downtown area of the city. Finally, the residential attractors are the least influential attractors in the morning period of typical weekdays. They are mostly located in the outer places of the city.

We further show what makes different regions in the city have different attraction profiles by relating types of places (POIs) to a region’s attraction behavior. To accomplish that, we used statistical significance testing to relate the decomposition of POI types (services) and the discovered attraction patterns. The statistcial significance testing approach allows us to quantify the signficance of each type of POI to each pattern of attractors discovered. We discuss each type of attractors and thier related POIs in the follwing sections.

#### Global attractors

**Fig 6:**
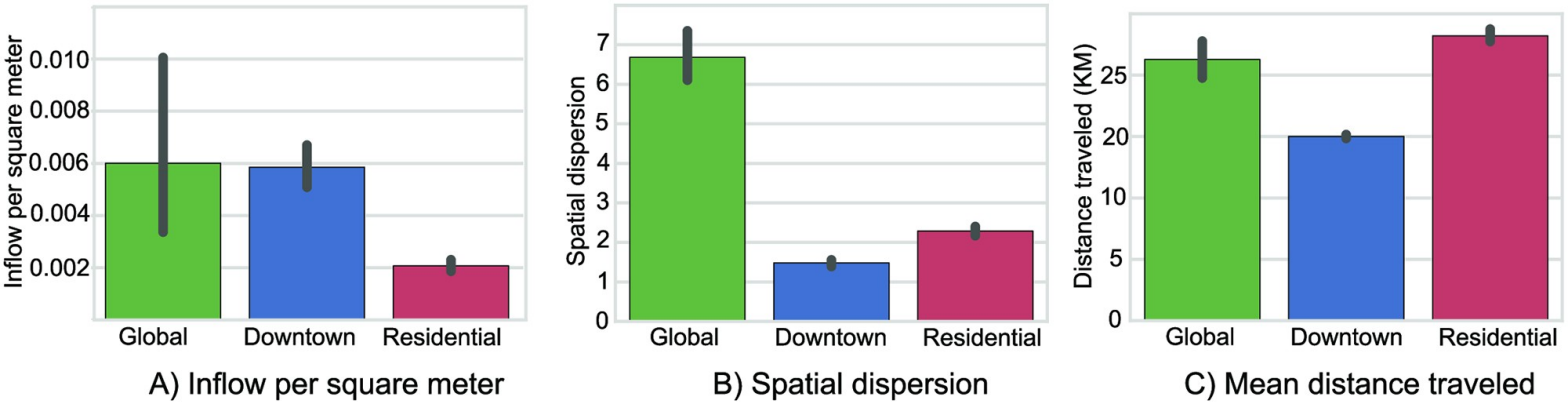
The attraction features of the three detected types of attractors clusters. (A) shows the total inflow, which is high for both global and downtown attractors but extremely low for residential attractors. (B) shows the spatial dispersion of origins of visitors, where it’s significantly high in global attractors. (C) shows the mean of the distance traveled by visitors, where downtown attractors exhibit smaller distance mean due to its central location and thus higher accessibility to most visitors.

We call these places global attractors because they strongly influence human mobility over the whole city. The most distinguishable feature of global attractors is the large spatial dispersion of the incoming flows, as visitors come from all over the city to visit these places, as shown in Fig 6*B*. Additionally, the amount of visitors they attract is the largest, as shown in Fig 6*A*, where we use inflow per square meter due to the unbalanced sizes of TAZes. Moreover, the mean distances traveled by visitors to these locations is extremely high (exceeding 20 KM), which makes these places highly attractive and unique.

Global attractors offer unique *services* that make them distinguished from other regions where visitors can only find such services in those regions. Some of the significant places occupy entire TAZes on their own and are easy to identify from the map, such as airports, major universities, and hospitals. Fig 5*A* shows the annotation of some of the major places identified as global attractors in the city of Riyadh.

**Fig 7:**
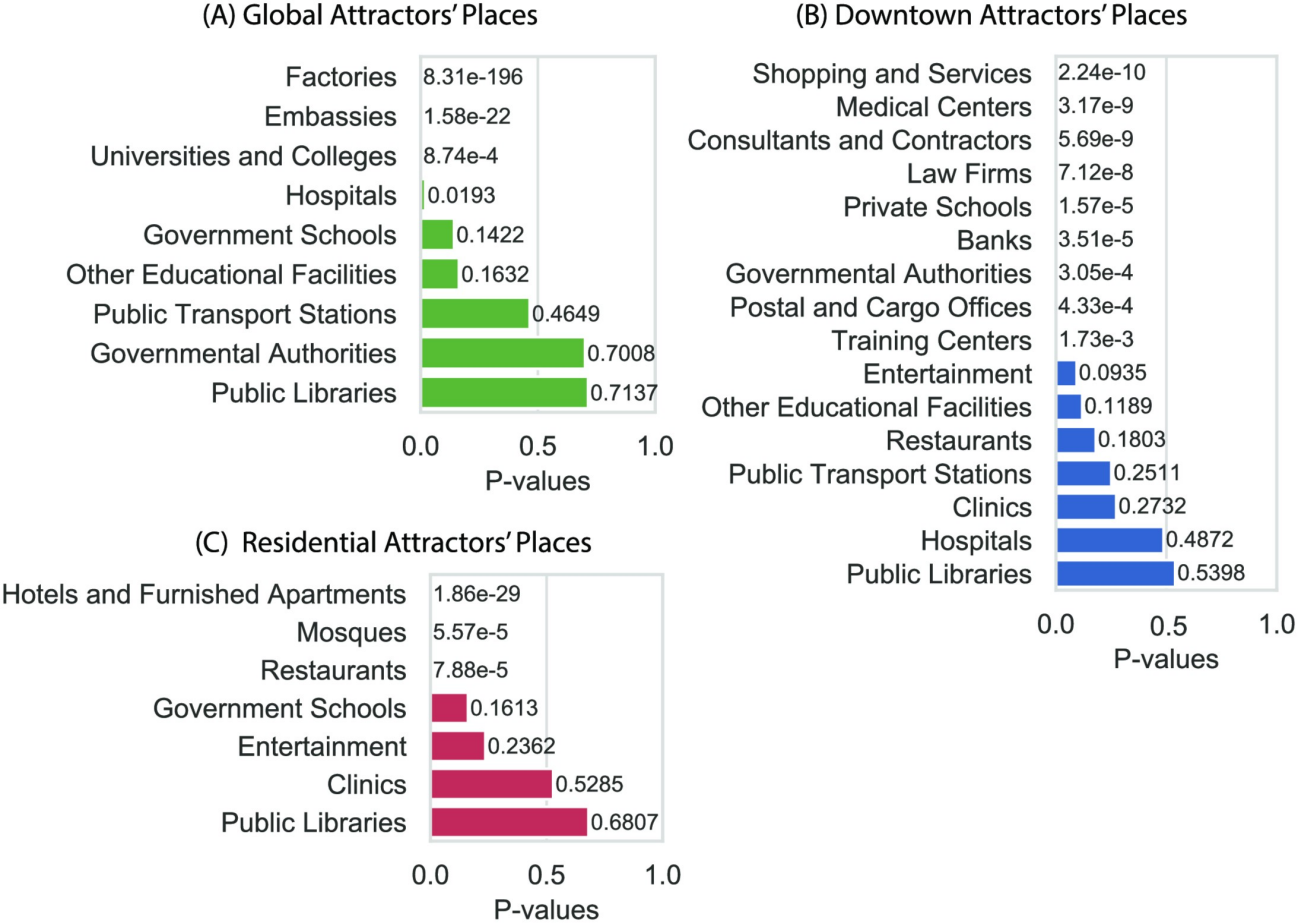
The types of POIs ordered by their statistical significance in the corresponding attractor’s type. (A) The p-values of finding each of the POI types in TAZes classified as global attractors, showing that factories, embassies, universities, and hospitals are type of services significantly located in global attractors. (B) POI types that are significant for the downtown attractors, which mostly includes business types and services. (C) POI types significantly present in the residential attractors that includes services and amenities such as furnished apartments, mosques (places of worship for Muslims), and public schools.

The POI types that are found to be significantly concentrated in global attractors are shown in Fig 7*A*, where each type of POIs ordered by their significance using their p-values. Factories, embassies, universities, and hospitals are the top POI types that attract massive amounts of people from all over the city. The city has three major universities attracting a student body of 40*k* each contributing significantly to the observed global attraction. Riyadh also has a major industrial area in the south to where a major number of factory workers commute. In addition, Riyadh city has the diplomatic quarter district, which hosts over 50 embassies attracting workers and visitors from all over the city to process documents. Also, Hospitals are expected to cause global attraction as visitors travel long distances from all over the city to reach them.

#### Downtown attractors

The second type of attractors is the downtown attractors, shown in Fig 5*B*. They contain regions that are mostly clustered in the central business district and are highly accessible. These are TAZes that have relatively high inflow, as shown in Fig 6*A*. However, because of their central location in the city, visitors from all over the city have short routes to access these places. They have smaller average distances compared to the other two types, as shown in Fig 6*C*. For the same reason, the dispersion of the origins from the center of mass of inflows is also small, as shown in Fig 6*B*. The significant feature of these places is that they attract a great number of visitors and are accessible.

Fig 7B shows the types of POIs mostly located in the downtown attractors in a descendant order by significance. We observe a large number of POI types with strong significance (p-values<0.01) compared to the other two types of attractors. That is due to the richness in quantity and variation of types of POIs in this area. Thus, we expect a larger number of POI types to be significant in these areas. The common theme of POI types in this attractor is businesses, which is typical in downtown regions.

#### Residential attractors

Residential attractors, which are shown in Fig 5*C* attract the smallest number of visitors in the morning hours of typical weekdays. As they are located in the outer sides of the city, visitors to these places travel long distances on average to reach them, as shown in Fig 6*C*. For the same reason, the dispersion of the small number of visitors is larger than the downtown attractors, as shown in Fig 6*B*.

Fig 7*C* shows the types of POIs concentrated in the residential attractors in a descendant order. The most significant types of POIs are services typically located in residential areas like furnished apartments, mosques (places of worship for Muslims), restaurants, including small restaurants and fast food places, and public schools. These places are not unique, so each residential neighborhood has its own share of such services to serve the population living nearby.

## Discussion

We presented a novel computational framework to discover different attraction patterns in cities. Our work contributes to understanding attractions of cities’ districts using attraction features that previous research did not cover. Previous research aimed to use mobility data to define districts’ function or land use in cities by features based on districts’ structure, characteristics such as services, and visitors’ demographics. In this work, we contribute to understanding districts’ attraction to visitors using features that define the attraction of urban zones. The features we proposed include: total number of incoming trips, the spatial dispersion of the origins of trips, and the distribution of distances traveled by visitors to reach that district. Further, we presented a method for understanding the relationship between the decomposition of POI types in a spatial zone and its attraction behavior. We applied the proposed method and showed results for the city of Riyadh, the capital of Saudi Arabia.

The results of implementing the proposed modules show interesting attraction patterns discovered. We detect three attraction patterns in the city of Riyadh according to the morning mobility dynamics. Global attractors represent districts that receive a large share of visitors traveling longer distances and coming from all over the city. These attractors have places of interest that are essential to visitors that include universities, factories, hospitals, embassies, and government facilities. The second type of attractors is that of the downtown areas, which receive a high inflow of people traveling shorter distances and with smaller spatial dispersion due to the central location of these districts in the city that makes them accessible. The most significant POIs types located in the downtown attractors are business based places like firms, shopping and service places. The least significant attractors are the residential areas in the morning hours, where the amount of inflow is the lowest. Residential attractors contain common POIs that serve residential neighborhoods such as apartments, mosques, and schools.

The proposed framework relies on mobility data mined from cell phones using the state of the art methods of extracting OD matrices [25]. The method used is validated against travel surveys and shown to have high accuracy. The extracted OD matrices provide a coherent understanding of the dynamics of the interaction between the flows of people to a district. OD matrices based on CDRs capture the mobility of a representative sample of the city’s population. Thus, they provide the best data coverage compared to other sources of data to capture mobility such as GPS, geo-tagged social media posts or checkins, where data biases is a large issue. Additionally, OD matrices preserve the privacy of cell phone users, since the trips are aggregated on the level of spatial zones (TAZes). This is a big advantage over GPS data, where privacy is a serious concern and is the primary reason for limited coverage. This advantage allows researchers to capture high coverage of trips and develop generalizable methods, while preserving the privacy of users’ data.

The proposed framework including the attraction features is easy to generalize to other cities to discover attractor patterns. This is because the framework relies on the OD matrix, which is a standard form of capturing mobility data in cities, and POI data which is vastly available publicly either from official cities autorites or from third party service companies such as Google Places, or Foursquare. Applying the framework using a different OD matrix and POI data is straightforward but researchers will have to tune the number of clusters using the elbow method we used to infer the attractors in the targeted city. While the method is generalizable, we might expect to discover different attractor patterns in different cities. Research [27] shows that cities lie on a spectrum of organization in terms of the distributions of hotspot districts inferred from mobility. This diversity in cities’ structrues is expected to depcit diverse attraction patterns. The diverse structures of cities are influenced by urban factors such as population mixing, transportation systems, and pollution. Whether the city is mono centric versus poly centric will likely affect patterns of its attractors behavior. Additionally, cities variation in services (POIs) distributions over districts is likely to influcne attractors behavior. For example, whether universities are located closer or further from the city center, and wheather disitricts are diverse or homogenous in POIs might result in different attraction behavior. Moreover, public transit systems structure is likely to influence accessibility and therefore attraction of districts.

One limitation in the proposed features is in using the distance instead of using the travel time to capture districts’ attraction. We recognize that travel time is a more accurate measure of attraction in congested cities. Computing travel time requires simulating congestion with CDRs. While this is possible [25], however, developing the congestion simulations requires substantial effort and can be done in future work. Thus, we proposed using the road network distances as an approximate alternative, assuming congestion does not influence the attraction.

Several interesting directions can follow this work. One is to compare attractions across different cities and examine whether there are common patterns. Also, to relate urban aspects of cities to attraction patterns such as placement of major POIs such as universities and hospitals, mono centricity versus poly centricity, and public transit systems versus relying on the road network. Another future direction is to examine attractors’ behavior using different time windows of the day and explore how attractors change in behavior temporally.

## Appendix

### Origin-destination matrix extraction

Our primary source of data to infer OD matrices is one month of Call Detail Records (CDRs) of anonymous mobile phone users in the city of Riyadh, Saudi Arabia. The data was collected by the mobile phone provider by billing and operational purposes that includes research and development agreements. For the researchers, this data is human subjects exempt via IRB training and approval.

**Fig 8:**
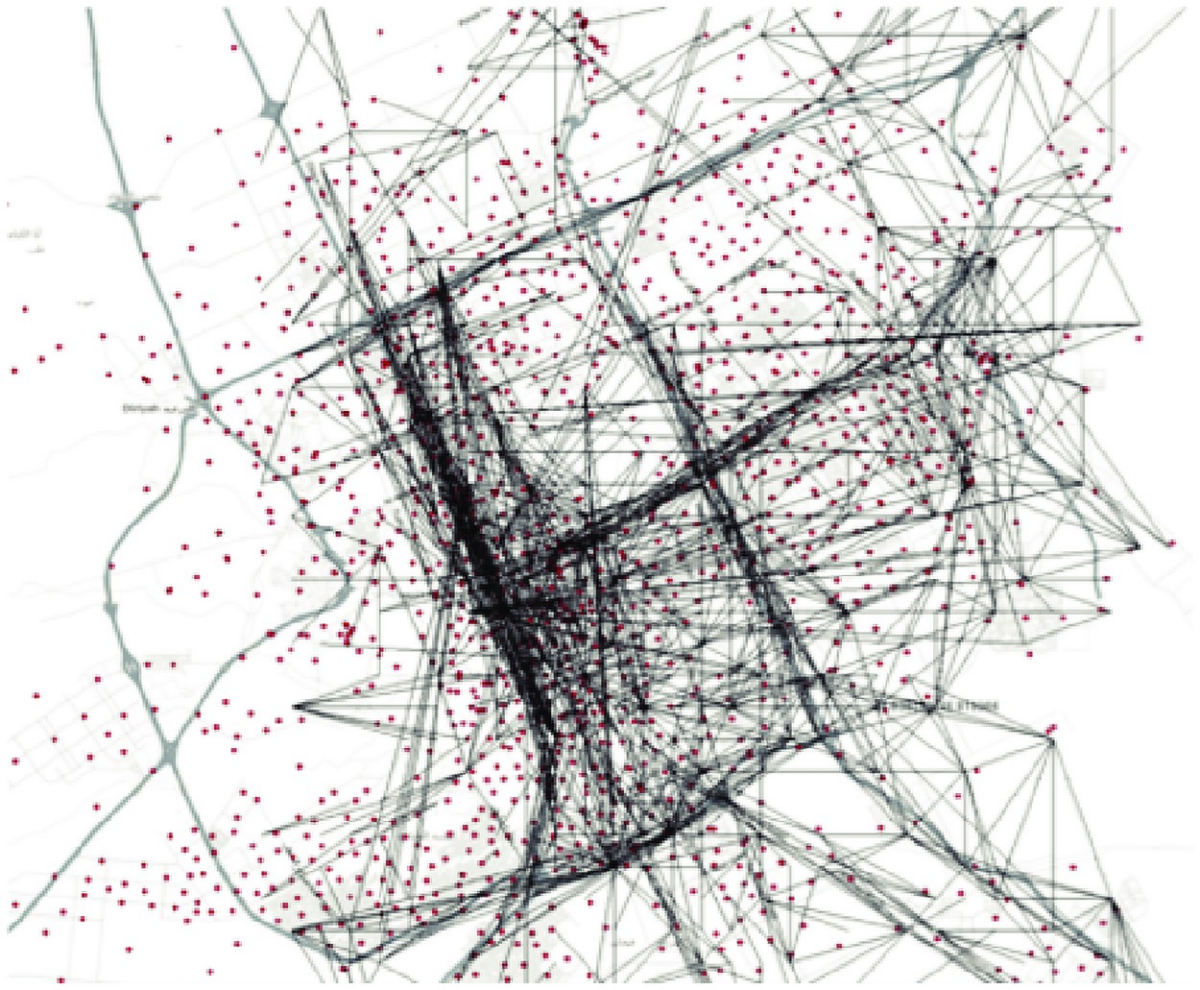
The ODs in Riyadh during the morning period. Each line represents a trip from a source to a destination.

The aim of this process is to extract the Origin-Destination matrices (ODs), that provides the number of trips between each pair of locations in the city for a specified time window of a typical weekday. Methods of estimating ODs range from traditional methods to more modern ones. In this paper, we use state of the art methods [5, 25] of extracting OD matrices for the city of Riyadh between each pair of traffic analysis zones (TAZes) as shown in Fig 8. The method is based on mobile phone location traces (i.e. CDRs) to estimate the flows of people between areas in the city. The large scale of the cell phone data provide sufficient sample sizes and more accurate information compared to traditional methods. They overcome the shortage in traditional methods such as running surveys within cities and estimating the flows between locations of the city from the feedback of those surveys. Such traditional methods consume longer periods of time and are inaccurate at times. They usually span smaller population sample sizes and thus are more prone to biases.

Within the CDRs, mobility is captured on the level of the cell tower facilitating the service. Each cell tower ID is spatially mapped to its latitude and longitude, where each Voronoi cell in Fig 8 correspond to a tower. The CDRs data contains more than 3 million unique users, which is a representative sample of Riyadh’s population. All users’ trips were anonymized and aggregated. No individual user data was ever manually inspected; only aggregated flows of large populations were handled. Thus, the CDRs provide a good proxy for tracking human mobility behavior in the city while preserving users privacy.

We follow the computational steps described [25] to transform raw mobile phone data (CDRs) to ODs. It can be summarized as follows. First, we estimate stay points of users’ trips by aggregating trajectory points in space and time (we use a threshold of 5 minutes). Then, we estimate daily trips from filtered users (with enough stay points) by analyzing consecutive observations at different stay points during a given time window. Then we scale from users to the total population using census data. The output of this process is the OD matrix that indicates the number of individuals traveling between every possible pair of locations between 7 am and 10 am on a typical weekday.

The spatial scale we used for locations is based on Traffic Analysis Zones (TAZes), which is the official segmentation used in transportation planning. Conventionally segmenting the city into TAZes are based on census block information such as population per hour, where zones tend to be smaller in denser areas and larger in areas of low density. The TAZ based segmentation is more flexible and useful in analyzing places attraction patterns than using other segmentations such as neighborhood based or spatially uniform segmentation. Thus, we define our OD matrix *T* by aggregating cell phone towers on 1492 TAZes. The elements of the matrix are the number of trips between each pair of TAZes (*i*,*j*) in the city.

### Robustness

#### Sensitivity to the similarity metric

To test the robustness of the framework proposed, we evaluated the sensitivity of results to the choice of the distance function used in Eq 7. We evaluated the clustering stability using methods described in [28].

We perform clustering using multiple similarity functions (listed in Table 1). Then, compared the labels that resulted from our chosen metric (correlation) *y* against labels resulted using other metrics $\hat{y}$. To be able to compare two sets of labels that are not necessarily in correspondence, the labels in one clustering results are permuted to maximize the overlap between the two clustering results. The clustering results comparison is measured using the normalized hamming distance defined as:

$$\begin{array}{c} d\left(y,\hat{y}\right)=\min_{\pi \in \psi }\frac{1}{n}\left\{1y\neq \pi (\hat{y})\right\}, \end{array}$$

where *π* is the permutation from the set of all permutations *ψ* that creates the largest overlap between *y* and $\hat{y}$. The results if using correlation and using other similarity functions are reported in Table 1. We observe that the results are not sensitive to the choice of the similarity function where only [1–10]% of districts differed in the classification identity. We observe high similarity results of using cosine distance, since the correlation distance is equivalent to the centered cosine distance function.

**Table 1:** Clustering stability measured by the hamming distance $d(y,\hat{y})$ of the clustering results of using the selected correlation metric against results form the other distance functions.

Distance	Definition	$d(y,\hat{y})$
Cosine	$1-\frac{x.y}{{∥x∥}_{2}.{∥y∥}_{2}}$	0.01
Euclidean	$\sqrt{\Sigma_{i}^{n}{\left(x_{i}-y_{i}\right)}^{2}}$	0.07
Manhattan	$\sqrt{\Sigma_{i}^{n}\left|x_{i}-y_{i}\right|}$	0.1
Minkowski	$\sqrt{\Sigma_{i}^{n}{|x_{i}-y_{i}|}^{2}}$	0.07
